# Vascular rejection in cardiac allograft vasculopathy: Impact on graft survival

**DOI:** 10.3389/fcvm.2022.919036

**Published:** 2022-08-04

**Authors:** Nandini Nair

**Affiliations:** Division of Cardiology, Department of Medicine, Texas Tech University Health Sciences Center (TTUHSC), Lubbock, TX, United States

**Keywords:** anti HLA antibodies, anti-vimentin antibodies, antibody-mediated rejection, donor specific antibodies, cardiac transplantation

## Introduction

In the more recent years antibody mediated rejection (AMR) which is a major contributor to vascular rejection has evolved as the most important cause of morbidity and mortality in patients undergoing cardiac transplantation. Acute AMR in the first year would be concerning but a long term consequence of chronic AMR is cardiac allograft vasculopathy (CAV) which has devastating consequences accounting for ~60% mortality (1–5).

AMR results largely from development of antibodies to the human leukocyte antigen (HLA classes I and II). Non-HLA antibodies have also been implicated in AMR in solid organ transplant recipients (6–8). Allosensitization can be due to a variety of causes including multiple blood transfusions, prior transplantation, human tissue allografts, and more recently the advent of durable mechanical circulatory devices. Prevalence of allosensitization has been noted in 30–50% women with >3 pregnancies due to pregnancy associated paternal antigens (9–13). This article addresses the impact of AMR, its consequences on survival of cardiac allograft recipients and the role of screening for donor specific antibodies (DSA), anti HLA antibodies as well as non-HLA antibodies in improving outcomes in this population.

## Pathophysiological mechanisms underlying graft destruction during AMR

The pathophysiology appears to be largely driven by three major components- non-HLA antibodies, anti HLA antibodies as well as donor specific antibodies. The specific role of each of these antibodies in the pathophysiology is discussed below.

## Non-HLA antibodies

The role of non-HLA antibodies in the pathogenesis of AMR remains less defined with limited data on their clinical significance, as well as pathophysiology of graft injury. A defined screening protocol for these antibodies should be executed post transplantation.

Non-HLA antibodies have been found to be directed against intracellular antigens. One of the mechanisms of graft destruction by non-HLA antibodies has been suggested *via* destruction of endothelial cells by DSA causing exposure of normally unseen autoantigens leading to production of non-HLA antibodies (14, 15).

Durable mechanical support could possibly trigger non-HLA antibodies. Patients bridged to transplant show higher serum reactivity. Such effects are also noted in patients who have undergone prior cardiac surgery and those with polyreactive antibodies (13, 16). It has been hypothesized that ventricular assist device (VAD) support causes polyreactive IgG antibodies directed to apoptotic cells and oxidized epitopes possibly due to non-specific B-cell activation and IgG sensitization and an inflammatory reaction concomitantly with PGD. Some of the antigens in particular that have been known to have antibodies in the AMR population are, vimentin, beta-tubulin, lamin A/C, and apolipoprotein L2. It is not clear how these mediate graft injury *via* their association with DSA during AMR (17).

It has also been noted that significant reactivity to non-HLA antigens was higher in patients who were positive for donor specific antibodies suggesting an association and synergy between the different types of antibodies. Elevated levels of polyreactive IgG antibodies have been associated with primary graft dysfunction (PGD) (16).

Patients diagnosed with AMR have recently been shown to have a reactive response to vimentin, beta-tubulin, lamin A/C, and apolipoprotein L2. Such increased reactive response to non-HLA antigens has been associated with AMR graft failure in solid organ transplants (18–22).

In the current literature a number of non-HLA antibodies have been shown to be involved in AMR (18–23). Non-HLA antibodies associate both with PRAs as well as DSA (23, 24). Patients with panel reactive antigen (PRAs) >10% during AMR showed increased reactivity to non-HLA antigens as compared to those who had no DSA or PRA. Positive PRA (>10%) without DSA suggests that circulating non-donor-specific anti-HLA antibodies (NDSA) or antibodies to non-HLA antigens could be the reason behind AMR and its impact on poor graft survival (24).

Antivimentin antibodies (AVA) have been implicated in AMR in animal models as well as human solid organ recipients. AVA is commonly seen in patients with autoimmune conditions like lupus/rheumatoid arthritis. Though vimentin is a cytoplasmic intermediary filament protein derived from the mesenchyme noted in leukocytes, fibroblasts, and endothelial cells it is often seen on the cell surface of cells serving as an autoantigen and eliciting an immune response in the autoimmune disease state especially on apoptotic neutrophils and T cells (25–31). AVA has been noted in both renal and cardiac transplant patients and notably in patients who develop cardiac allograft vasculopathy (CAV) (19, 32). Studies show an increased numbers of IL-17 secreting CD4+ T cells directed at vimentin and a reduction in IL-10 producing cells in patients with CAV, suggestive of vanishing tolerance to vimentin in patients with CAV (19, 33). Another important aspect of AVA in cardiac transplant patients is their differential response to immunosuppressive drugs. Mycophenolate mofetil (MMF) has been found to better in reducing AVA as well as HLA antibodies (34). In non-human primates AVA levels were not affected by cyclosporine treatment showing its ineffectiveness in this respect (27). The deleterious effects of AVA possibly occurs *via* complement fixation and a proinflammatory effect. Additionally, the interaction of AVA with neutrophils can lead to platelet activation causing a prothrombotic effect in the graft vasculature.

Other notable associations have been between anti cytoskeletal anti endothelial cell antibodies and cardiac allograft rejection and those against agrin, adipocyte plasma membrane-associated protein, Rho GDP-dissociation inhibitor 2 [ARHGDIB], Rho guanine nucleotide exchange factor 6, angiotensin-II type 1 receptor, endothelin type A receptor, lamin B1, BPI fold-containing family B member 1, peroxisomal trans-2-enoyl-coenzyme A reductase, phospholipase A2 receptor, protein kinase C zeta type, tubulin beta-4B class IVb in renal transplant patients suggestive of a significant role for non-HLA antibodies in AMR in solid organ recipients (35, 36).

## Anti HLA antibodies

The activation of endothelial cells by anti HLA antibodies has been postulated to cause proliferation, cytokine production and leukocyte recruitment leading to graft injury and dysfunction. Checking pretransplant anti HLA antibodies should be done imperatively as part of the pre transplant work up algorithm because sensitization has been noted with increasing length of mechanical support. Interestingly, avoidance of perioperative leukocyte-filtered cellular blood product transfusions does not significantly decrease the incidence or degree of HLA sensitization. However, cellular blood product transfusions have been noted in some studies to reduce the possibility of alloimmunization which may reduce the problem in the patients bridged to transplantation (16, 17, 37).

The critical role of anti HLA antibodies in the pathogenesis of AMR and its ultimate impact on allograft survival leads credence for active surveillance of these antibodies pre and post transplantation. The anti HLA antibodies work *via* multifactorial mechanisms to destroy the graft. The most important of these is the complement activation cascade and formation of the membrane attack complex (MAC). Early proinflammatory proteins have recently been shown to drive the complement mediated injury. Other mechanisms include those mediated by the natural killer (NK) cells (38). Mechanisms independent of the complement system such as antibody-dependent cell-mediated toxicity (ADCC) utilize NK cells, neutrophils and macrophages which bind antibody-coated target cells *via* DC16 resulting in activation of effector immune cells. In NK cells, perforin and granzyme B-mediated cytotoxicity are noted while macrophages use nitric oxide, TNF, and reactive oxygen species to elicit cellular damage (39–42).

Gender differences in alloreactivity is an important aspect that needs to be taken into account when screening for anti HLA antibodies pre transplantation. Female cardiac allograft recipients have a higher pretransplant diagnosis of idiopathic cardiomyopathy, increase levels of antinuclear antibodies, in addition to HLA-B8, DR3 haplotypes. Female heart transplant patients tend to have a shorter duration to the first episode of rejection, increased number, and frequency of rejection episodes, and tend to produce anti-HLA antibodies sooner than their male counterparts. Infection related mortality seem to be higher in female cardiac allograft recipients. Fatal infections have been in noted in female heart transplant patients due to increased cyclosporine levels. Another interesting observation has been that the incidence of CAV developing after the first year post-transplant was lower in females. Women tend to manifest an autoimmune state prior to transplant which may predispose them to a greater risk of allograft. An algorithm for early diagnosis and management including a more individualized approach will improve clinical outcomes in the female population (9).

## Donor specific antibodies

DSA play a major role in development of AMR leading to loss of graft function and survival (3, 43–45). Presence of DSA are linked to increased graft loss and mortality. Interestingly preexisting DSA do not seem to affect survival significantly but *de-novo* DSAs (especially Class II) are associated with worse outcomes (46–50). Regardless of when DSA appear, early on or later, it confers a negative impact on graft outcomes. One of the important aspects of DSA is that they can escape detection from circulation because they can adhere to the graft and be just as detrimental. Presence of DSA can predict worse outcomes for the graft in the long term (51–54). AMR has been associated with CAV and presence of DSA has been associated with more severe CAV (55). DSA has been shown to cause endothelial activation leading to allograft dysfunction. Three to eleven percent of patients exhibit the presence of DSA at heart transplantation, interestingly *de novo* anti HLA II DSA seem to develop post-transplant in 10–30% of patients.

## AMR and CAV

The role of AMR in pathogenesis of CAV is becoming more evident. AMR is frequently noted patients with CAV in grafts and asymptomatic AMR is associated with a higher risk for CAV and increased mortality. CAV is a progressive obliterative disease due to intimal proliferation and most often not amenable to percutaneous coronary interventions or cardiac surgical revascularization. It has been estimated that >10% of adult cardiac transplant patients carry a diagnosis of CAV at 1 year and >50% at 10 years post transplantation (55–58). The major cause of CAV is alloimmunity with multiple factors including non-immunological attributes of the donor and recipient such as age and medical comorbidities.

AMR and CAV have been increasingly associated, with AMR occurring more frequently in patients with CAV. Patients with asymptomatic AMR show higher risk for CAV. In addition to DSA involvement in AMR induced CAV direct role of complement activation also plays a significant role. Endothelial cell cells get damaged directly due to C4d complement activation and deposition. Targeting of NK cells and macrophages can lead to progression of plaque formation. DSA and complement activation in combination affect endothelial cells, platelets, and macrophages to accentuate the progression of CAV (59).

## Discussion

**Figure 1:**
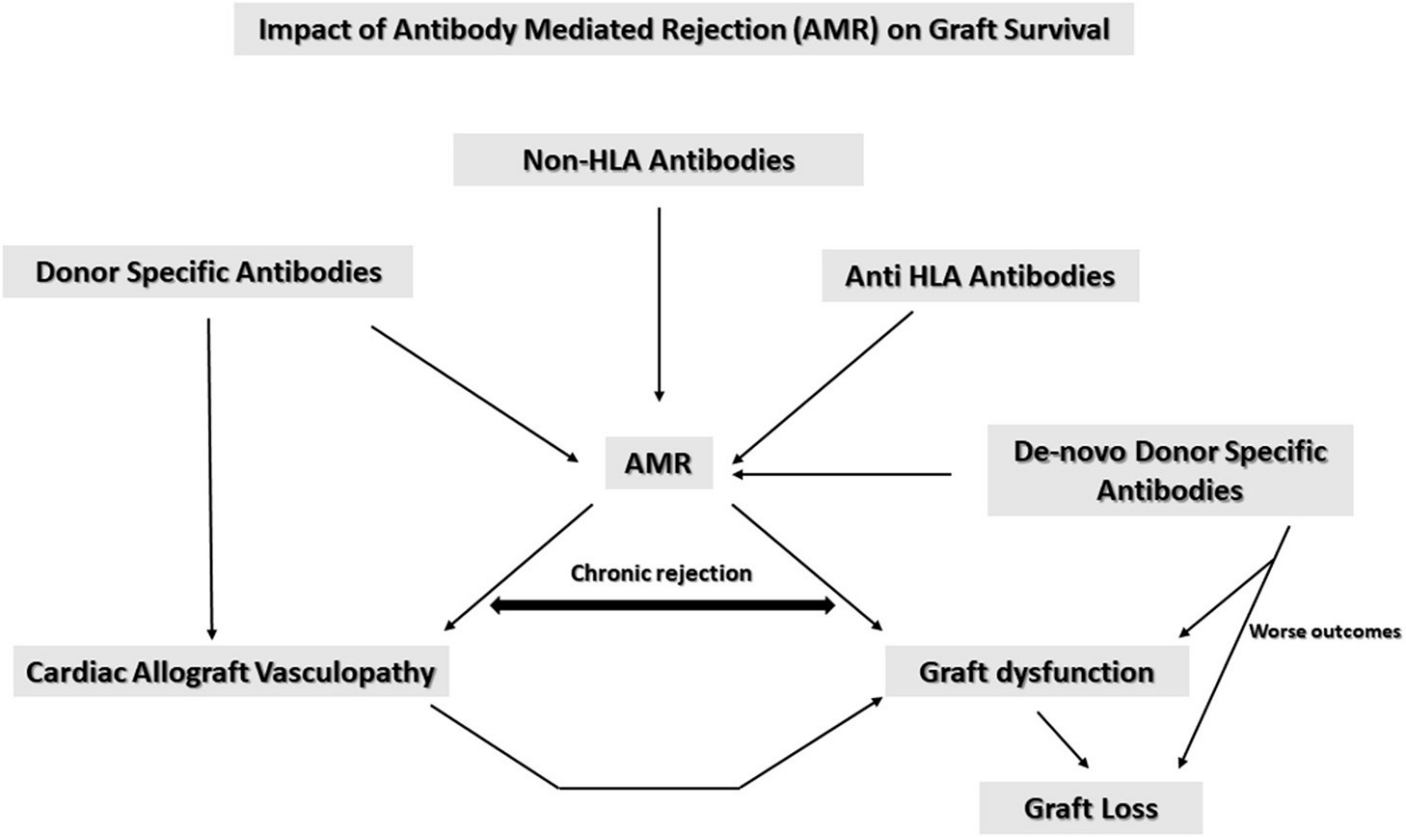
Impact of antibody mediated rejection on graft survival.

In summary AMR is a primary cause for graft dysfunction and mortality. As summarized in Figure 1 anti HLA antibodies, non-HLA antibodies and DSA all contribute to AMR. AMR also is associated with CAV which in turn causes long term graft dysfunction and graft loss leading to significant morbidity and mortality. DSA are associated with CAV risk. Patients with anti HLA class II antibodies have a shorter time to CAV diagnosis (4 years approximately) vs. those with only anti HLA class 1 antibodies (7–8 years approximately). Patients with a mixture of antibodies to both classes I and II develop CAV within 2 years and are therefore at the highest risk (43).

Newer technologies using molecular biology techniques such as targeted amplification of donor-derived cell free DNA (dd-cfDNA) may be the beginning of a highly sensitive method of detecting AMR even before it can be detected by endomyocardial biopsy which is the current method of diagnosis. This technology is based on the premise that in the setting of acute rejection allograft cell death occurs resulting in elevated levels of dd-cfDNA in the recipient's circulation (60, 61). Such efforts to detect subclinical injury will help early diagnosis of AMR before possible graft injury. Application of molecular technologies for early clinical diagnosis may be the future for graft preservation.

Current literature attributes DSA a major role in determining the fate of the transplanted heart. In the past, DSA considered harmful were essentially those directed against ABO blood group antigens or donor HLA antigens. More recent literature shows antibodies directed against other molecules expressed by the donor organ such as anti-endothelial cell antibodies or antibodies against MHC Class I-related chain A (MICA), anti-endothelin-1 receptor type A (ETAR), Perlecan and anti-angiotensin II receptor type 1 (AT1R) are equally relevant. In addition to IgG, IgM is associated with worse graft outcomes in the long term. Additionally, DSA that are capable of activating the complement system produce worse outcomes than DSA which act *via* a complement-independent mechanism. Contrary to the earlier hypothesis *de-novo* are also associated with poorer outcomes. Currently patients with non-significantly low levels of DSA have been postulated to be safe to transplant with closer follow up of antibody levels which is becoming more feasible with new molecular technologies such as the use of dd-cfDNA (39, 62–67).

Monitoring for DSA and *de novo* DSA post-transplant could provide an early diagnostic strategy for early intervention and better outcomes for graft protection. However, due to lack of existing studies definitive algorithms for surveillance and treatment are non-existent. The surveillance strategies and treatment protocols vary from center to center. More studies are needed at a multicenter level to assess antibodies at pre and post-transplant at different time points to develop more definitive protocols for surveillance and treatment. Building registries would help with defining thresholds for intervention and modifying peri operative management. AMR still remains a challenge and largely responsible for graft loss in the present era. Definitive studies are required in this area to improve outcomes and graft dysfunction and loss.

## Author contributions

NN conceived the idea and prepared the entire manuscript.

## Conflict of interest

The author declares that the research was conducted in the absence of any commercial or financial relationships that could be construed as a potential conflict of interest.

## Publisher's note

All claims expressed in this article are solely those of the authors and do not necessarily represent those of their affiliated organizations, or those of the publisher, the editors and the reviewers. Any product that may be evaluated in this article, or claim that may be made by its manufacturer, is not guaranteed or endorsed by the publisher.
